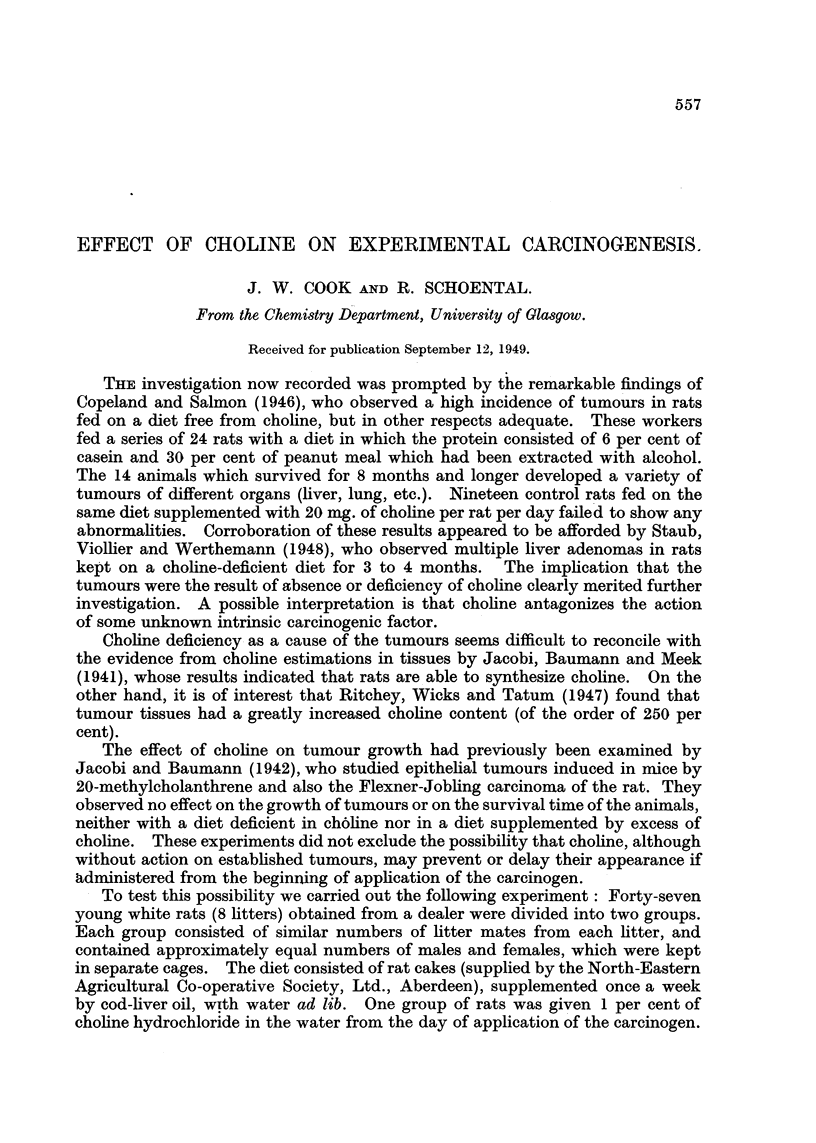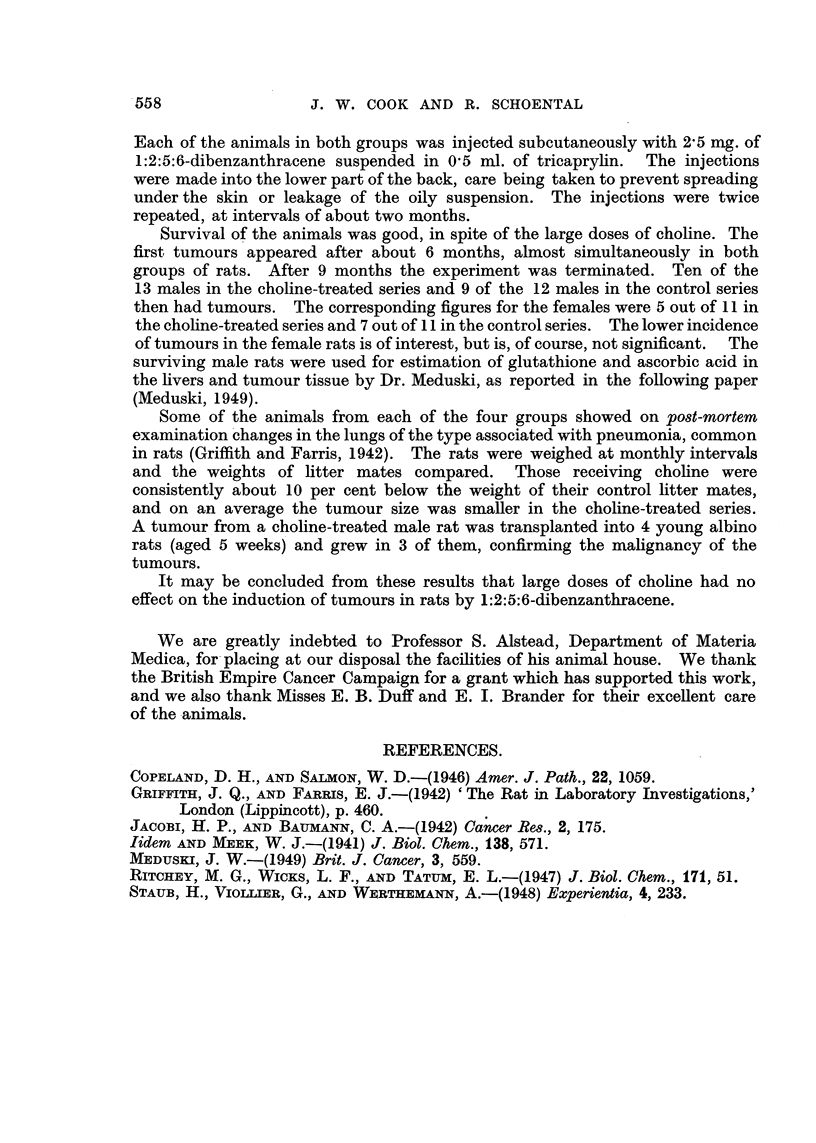# Effect of Choline on Experimental Carcinogenesis

**DOI:** 10.1038/bjc.1949.61

**Published:** 1949-12

**Authors:** J. W. Cook, R. Schoental


### EFFECT OF CHOLINE ON EXPERIMENTAL CARCINOGENESIS.

J. W. COOK AND R. SCHOENTAL.

*From the Chemistry Department, University of Glasgow.*

Received for publication September 12, 1949.

THE investigation now recorded was prompted by the remarkable findings of Copeland and Salmon (1946), who observed a high incidence of tumours in rats fed on a diet free from choline, but in other respects adequate. These workers fed a series of 24 rats with a diet in which the protein consisted of 6 per cent of casein and 30 per cent of peanut meal which had been extracted with alcohol. The 14 animals which survived for 8 months and longer developed a variety of tumours of different organs (liver, lung, etc.). Nineteen control rats fed on the same diet supplemented with 20 mg. of choline per rat per day failed to show any abnormalities. Corroboration of these results appeared to be afforded by Staub, Viollier and Werthemann (1948), who observed multiple liver adenomas in rats kept on a choline-deficient diet for 3 to 4 months. The implication that the tumours were the result of absence or deficiency of choline clearly merited further investigation. A possible interpretation is that choline antagonizes the action of some unknown intrinsic carcinogenic factor.

Choline deficiency as a cause of the tumours seems difficult to reconcile with the evidence from choline estimations in tissues by Jacobi, Baumann and Meek (1941), whose results indicated that rats are able to synthesize choline. On the other hand, it is of interest that Ritchey, Wicks and Tatum (1947) found that tumour tissues had a greatly increased choline content (of the order of 250 per cent).

The effect of choline on tumour growth had previously been examined by Jacobi and Baumann (1942), who studied epithelial tumours induced in mice by 20-methylcholanthrene and also the Flexner-Jobling carcinoma of the rat. They observed no effect on the growth of tumours or on the survival time of the animals, neither with a diet deficient in chôline nor in a diet supplemented by excess of choline. These experiments did not exclude the possibility that choline, although without action on established tumours, may prevent or delay their appearance if administered from the beginning of application of the carcinogen.

To test this possibility we carried out the following experiment: Forty-seven young white rats (8 litters) obtained from a dealer were divided into two groups. Each group consisted of similar numbers of litter mates from each litter, and contained approximately equal numbers of males and females, which were kept in separate cages. The diet consisted of rat cakes (supplied by the North-Eastern Agricultural Co-operative Society, Ltd., Aberdeen), supplemented once a week by cod-liver oil, with water *ad lib.* One group of rats was given 1 per cent of choline hydrochloride in the water from the day of application of the carcinogen.

Each of the animals in both groups was injected subcutaneously with 2·5 mg. of 1:2:5:6-dibenzanthracene suspended in 0·5 ml. of tricaprylin. The injections were made into the lower part of the back, care being taken to prevent spreading under the skin or leakage of the oily suspension. The injections were twice repeated, at intervals of about two months.

Survival of the animals was good, in spite of the large doses of choline. The first tumours appeared after about 6 months, almost simultaneously in both groups of rats. After 9 months the experiment was terminated. Ten of the 13 males in the choline-treated series and 9 of the 12 males in the control series then had tumours. The corresponding figures for the females were 5 out of 11 in the choline-treated series and 7 out of 11 in the control series. The lower incidence of tumours in the female rats is of interest, but is, of course, not significant. The surviving male rats were used for estimation of glutathione and ascorbic acid in the livers and tumour tissue by Dr. Meduski, as reported in the following paper (Meduski, 1949).

Some of the animals from each of the four groups showed on *post-mortem* examination changes in the lungs of the type associated with pneumonia, common in rats (Griffith and Farris, 1942). The rats were weighed at monthly intervals and the weights of litter mates compared. Those receiving choline were consistently about 10 per cent below the weight of their control litter mates, and on an average the tumour size was smaller in the choline-treated series. A tumour from a choline-treated male rat was transplanted into 4 young albino rats (aged 5 weeks) and grew in 3 of them, confirming the malignancy of the tumours.

It may be concluded from these results that large doses of choline had no effect on the induction of tumours in rats by 1:2:5:6-dibenzanthracene.

We are greatly indebted to Professor S. Alstead, Department of Materia Medica, for placing at our disposal the facilities of his animal house. We thank the British Empire Cancer Campaign for a grant which has supported this work, and we also thank Misses E. B. Duff and E. I. Brander for their excellent care of the animals.

### REFERENCES.

Copeland, D. H., and Salmon, W. D.—(1946) *Amer. J. Path.*, **22**, 1059.
Griffith, J. Q., and Farris, E. J.—(1942) 'The Rat in Laboratory Investigations,' London (Lippincott), p. 460.
Jacobi, H. P., and Baumann, C. A.—(1942) *Cancer Res.*, **2**, 175.
*Iidem* and Meek, W. J.—(1941) *J. Biol. Chem.*, **138**, 571.
Meduski, J. W.—(1949) *Brit. J. Cancer*, **3**, 559.
Ritchey, M. G., Wicks, L. F., and Tatum, E. L.—(1947) *J. Biol. Chem.*, **171**, 51.
Staub, H., Viollier, G., and Werthemann, A.—(1948) *Experientia*, **4**, 233.